# Characteristics of Hybrid Bioglass-Chitosan Coatings on the Plasma Activated PEEK Polymer

**DOI:** 10.3390/molecules28041729

**Published:** 2023-02-11

**Authors:** Kacper Przykaza, Małgorzata Jurak, Grzegorz Kalisz, Robert Mroczka, Agnieszka Ewa Wiącek

**Affiliations:** 1 Department of Interfacial Phenomena, Institute of Chemical Sciences, Faculty of Chemistry, Maria Curie-Sklodowska University, Maria Curie-Sklodowska Sq. 3, 20-031 Lublin, Poland; 2 Department of Bioanalytics, Faculty of Biomedicine, Medical University of Lublin, Jaczewskiego St. 8b, 20-090 Lublin, Poland; 3 Independent Unit of Spectroscopy and Chemical Imaging, Medical University of Lublin, Chodzki St. 4a, 20-093 Lublin, Poland; 4 Laboratory of X-ray Optics, Centre for Interdisciplinary Research, The John Paul II Catholic University of Lublin, Konstantynow St. 1J, 20-708 Lublin, Poland

**Keywords:** PEEK polymer, chitosan, bioglass, biocompatible composite

## Abstract

Polyetheretherketone (PEEK) is a biocompatible, chemically and physically stable radiolucent polymer that exhibits a similar elastic modulus to the normal human bone, making it an attractive orthopedic implant material. However, PEEK is biologically inert, preventing strong enough bonding with the surrounding bone tissue when implanted in vivo. Surface modification and composite preparation are the two main strategies for the improvement of the bioactivity of PEEK. In this study, the plasma activated PEEK surfaces with the embedded bioglass, chitosan, and bioglass-chitosan mixed layers applying from the solution dip-coating technique were investigated. The most prominent factors affecting the coating biocompatibility are strictly connected with the composition of its outer surface (its charge and functional groups), hydrophilic-hydrophobic character, wettability and surface free energy, and topography (size of pores/substructures, roughness, stiffness), as well as the personal characteristics of the patient. The obtained surfaces were examined in terms of wettability and surface-free energy changes. Additionally, FTIR (Fourier Transformation Infrared Spectrometry) and SIMS (Secondary Ion Mass Spectrometry) were applied to establish and control the coating composition. Simultaneously the structure of coatings was visualized with the aid of SEM (Scanning Electron Microscopy). Finally, the obtained systems were incubated in SBF (Simulated Body Fluid) to verify the modifications’ influence on the bioactivity/biocompatibility of the PEEK surface. Different structures with variable compositions, as well as changes of the wettability, were observed depending on the applied modification. In addition, the incubation in SBF suggested that the bioglass-chitosan ratio influenced the formation of apatite-like structures on the modified PEEK surfaces.

## 1. Introduction

Biocompatibility is an extremely important issue in terms of modern science, particularly in medicine, biotechnology, material, and tissue engineering. To this day, a huge challenge for scientists is to obtain an ideal material that is highly compatible with appropriate applications, e.g., as a bone substitute. Synthetic polymers often exhibit mechanical properties that can allow them to be applied in a specific area of medicine, however, the lack of desired response from the body frequently prevents their use. This effect is influenced by many factors, such as surface composition, degradation, topography, surface wettability, and its free energy, as well as competitive protein binding [1]. For example, the presence of appropriate functional groups on the surface of the modified material can promote cell adhesion and proliferation, and thus the tissue will grow onto the foreign body as if it were a product of a given organism. One of the promising polymeric materials is polyetheretherketone (PEEK), owing to its excellent mechanical strength, biocompatibility, chemical resistance, and radiolucency. Moreover, PEEK reveals no toxicity to human tissues, shows elastic modulus very close to human bone, and does not interfere with the postoperative X-ray or magnetic resonance imaging (MRI) [2]. That is why PEEK was already used for the orthopedic implants, such as spinal cages, skull plates, and joints [3,4]. Unfortunately, its integration with the bone tissue is highly limited, most likely due to the insufficient bioactivity of PEEK induced by the small surface free energy, hydrophobic character, lack of active groups, and, thus, inappropriate protein binding to the surface [5]. To overcome this problem, various chemical and physical modifications of the top polymer layer are used. For example: sandblasting [6], plasma treatment [7], acid etching [8], laser processing [9], and the deposition of bioactive substances, such as polysaccharides, sterols, peptides, phospholipids, hydroxyapatite, or titanium layers [4,10,11,12]. One of the biocompatible, degradable, and non-toxic polysaccharides is chitosan (Chit). This derivative of natural chitin gained significant popularity in many branches of science, especially in tissue engineering. Chitosan is characterized by antibacterial properties and stimulates proliferation and cell adhesion, thus accelerating the wound healing process [13].

The Bioactivity Index ($I_{B}$) of a material is the time taken for more than half of the interface to bind:

$$(t_{0.5bb})\;I_{B}=100/t_{0.5bb}.$$

The scale of $I_{B}$ starts with 0, which means no bioactivity, and goes up to 13 so far. The materials characterized by the $I_{B}$ index greater than 8 will bind to both the soft and hard tissues. These are called osteoinductive and are assigned to the class A biomaterials [14]. Their surface promotes the colonization of bone stem cells and induces their proliferation and differentiation. The examples of class A bioceramics are bioactive glasses and glass-ceramic. When the *I_B_* index is smaller than 8, the materials can be included in class B and are called osteoconductive, which means they elicit only an extracellular tissue response. The substances from the hydroxyapatite family are considered as the class B biomaterials [15].

Still, one of the most biocompatible materials ever discovered is bioglass (BG) (the in vivo bioactivity index $I_{B}$ is around 12.5). The most common BG material is the bioglass named 45S5. It was developed in 1969 by Larry Hench and its major components are: ${\mathrm{SiO}}_{2}$ (45% wt); ${\mathrm{Na}}_{2}O$ (24.5% wt); CaO (24.5% wt); and $P_{2}O_{5}$ (6% wt) [14]. Similar to chitosan, this material is established as degradable in the body and what is most important is that it promotes osteoblasts adhesion, migration, proliferation, and mineralization of extra cellular matrix (ECM) into the bone tissue. Thus, it is suitable for applications involving direct contact with the bone, however, due to its insufficient mechanical properties, only as a graft or coating. The combination of bioglass and chitosan as a coating on the PEEK surface was already the main topic of other studies [6,16,17]. For example, Hong et al. tested the BG-Chit coatings deposited on the sandblasted and acid etched PEEK surfaces obtained by the dip-coating technique and observed a potential increase in the material biocompatibility based on the in vitro tests [6]. The other group developed multilayered PEEK/BG containing chitosan/gelatin/Ag-Mn with the aid of electrophoretic deposition (EPD). They established a great bioactivity, applying immersion in SBF, as well as the antibacterial activity, against the Gram-positive and Gram-negative bacteria [18]. Rehman et al. described extensively the preparation of PEEK/BG coatings on the stainless steel and its further coverage with a layer of chitosan-drug applying the EPD technique, which improved the corrosion resistance, wetting properties, and allowed to release the drug from the coating [17]. The coatings based on bioglass and chitosan exhibited good physicochemical and mechanical properties, indicating their possible use in regenerative medicine. However, the substrate had to be previously modified to ensure the proper adhesion to the coating. These modifications are usually time-consuming and can lead to excessive surface smoothing or deterioration of its adhesive properties [6]. Considering the unique ability of the plasma treatment, which can largely enhance the adhesion properties of the treated surface, in our experiment we applied cold plasma activation of PEEK surface to develop the bioglass-chitosan hybrid coating. Combining this approach with the dip-coating technique, we deposited stable bioglass, chitosan, and bioglass-chitosan mixed layers successfully on the activated PEEK surface. The surface properties (morphology, chemistry, wetting) of the PEEK were significantly altered by these modifications. Additionally, we proposed a faster and less complicated approach compared with the EPD technique. Moreover, the impact of the amount of the chitosan on the surface properties in combination with the bioglass was determined. Indeed, the presence of bioglass and chitosan in the coating should improve the biocompatibility and induce antibacterial properties [17,19]. We believe that precise combination in terms of the composition and adhesive strength of bioglass and chitosan can lead to the invention of new hybrid materials, which can merge the biocompatibility and physicochemical properties of both components.

## 2. Results and Discussion

### 2.1. FTIR Analysis

**Figure 1:**
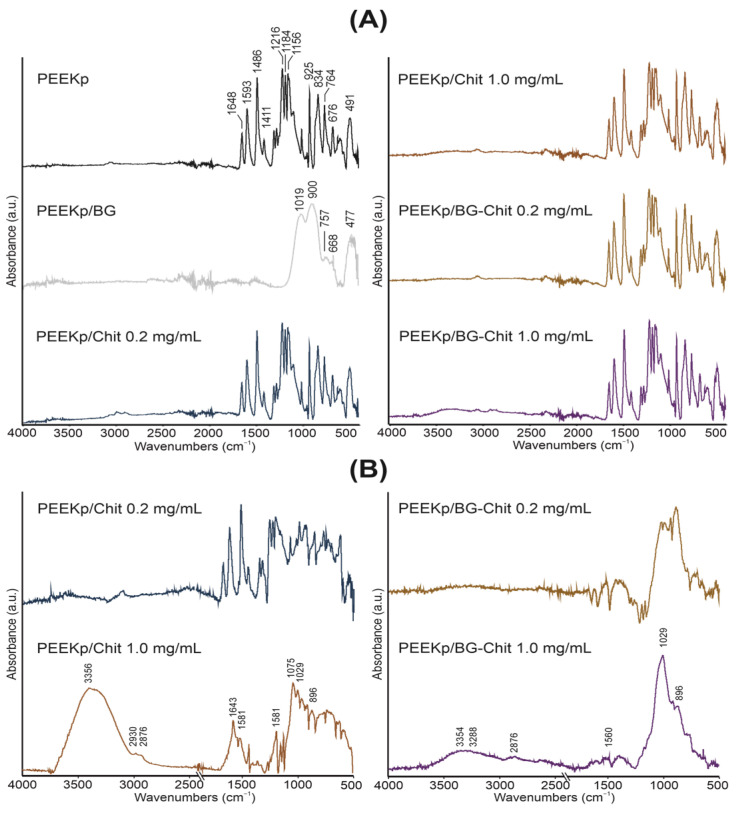
(**A**). FTIR spectra of the modified PEEK surfaces, normalized to the highest absorbance band. (**B**). FTIR subtraction spectra of PEEKp control material and chitosan-containing PEEKp surfaces.

The full spectra acquired from the investigated samples confirm the identification of chitosan and bioglass within the samples and are presented in Figure 1A. The control sample of the cold-plasma activated PEEK is consistent with the literature data [20,21].

The PEEK spectra reveal typical bands at 951, 834, 764, 676 cm^−1^ from the CH out- of- plane bending vibrations of an aromatic ring. The band at 926 cm^−1^ belongs to the Aryl−(C=O)−Aryl symmetric stretching vibrations, and the maxima of bands at 1593, 1486, 1411, and 1156 cm^−1^ are assigned to the benzene skeleton vibrations, asymmetric stretching and rocking. The band at 1648 cm^−1^ indicates C=O stretching and asymmetric stretching of C−O−C at 1216 and 1184 cm^−1^. The spectrum derived from bioglass is presented in Figure 1A. The maxima of bands at the 477 cm^−1^ peak are corresponding to Si−O−Si bending vibration in the $SiO_{4}^{4-}$ group, indicating the presence of amorphous silicate [22,23] and Si−O−Si asymmetric stretching at 1019 cm^−1^ [24]. The band located at 900 cm^−1^ come from the stretching of the non-bridging oxygen atoms in the Si−O bond [24]. The absorption of P−O bending vibrations is located at 757 cm^−1^, combined with the 668 cm^−1^ value, is attributed to the stretching vibrations of phosphate groups [25]. No peaks that can be assigned to the organic matter are observed, confirming the bioglass purity. To remove the strongly overlapping signal of PEEK in the experimental samples, the spectrum of the PEEKp is subtracted from the others. The results of the subtraction are presented in Figure 1B. The characteristic bands of chitosan can be found in PEEKp/Chit 1.0 mg/mL and PEEKp/BG−Chit 1.0 mg/mL with the visible bands at 3354 cm^−1^ and 3288 cm^−1^, and originate from the N−H stretching of primary amines and O−H stretching. The bands at 2876 cm^−1^ are due to the C−H symmetric stretching. The band at 1560 cm^−1^ was attributed to amide II (N−H), however, there were no observable bands from the amide I and amide III bonds [26,27]. Nevertheless, the amide I, II, and III bands not corresponding to those observed in PEEKp were detected in PEEKp/BG-Chit 1.0 mg/mL. The band around 1029 cm^−1^ is from the C–N stretching and the C–O stretching [28]. The bands at 896 and 1075 cm^−1^ were corresponding to the glycosidic C−O−C stretching of the cross-linked chitosan [27,29]. The above description correlates with the conclusions drawn by other authors. Applying the XPS technique, Wiącek et al. observed a significant increase in the C−O, C−N, and C−O−C groups, and a simultaneous decrease in the C−C group on the plasma activated PEEK surface covered with the chitosan coating [30].

At this point, despite the rich spectrum of the PEEK substrate and the additional signals from bioglass, many signals characteristic of the chitosan coating were successfully located and identified, but only for the high concentration of chitosan (1.0 mg/mL). In similar research by other authors, when PEEK was activated by the nitrogen plasma and the 0.1 mg/mL chitosan solution was spread, they observed only higher signal intensity in the range of 3500–3200 cm^−1^, without identification of specific peaks [31]. This is probably due to the insufficient sensitivity of the FTIR technique for the samples prepared from a solution with a lower concentration of chitosan (0.2 mg/mL), since its ions were detected when applying the much more sensitive TOF-SIMS analysis [11]. Nevertheless, a significant reduction in the intensity (depletion of the spectrum) of the chitosan signals after deposition with bioglass for the 1.0 mg/mL chitosan samples was observed and can be associated with the specific co-adsorption of both components, or the attenuation of one signal.

### 2.2. SEM Imaging

**Figure 2:**
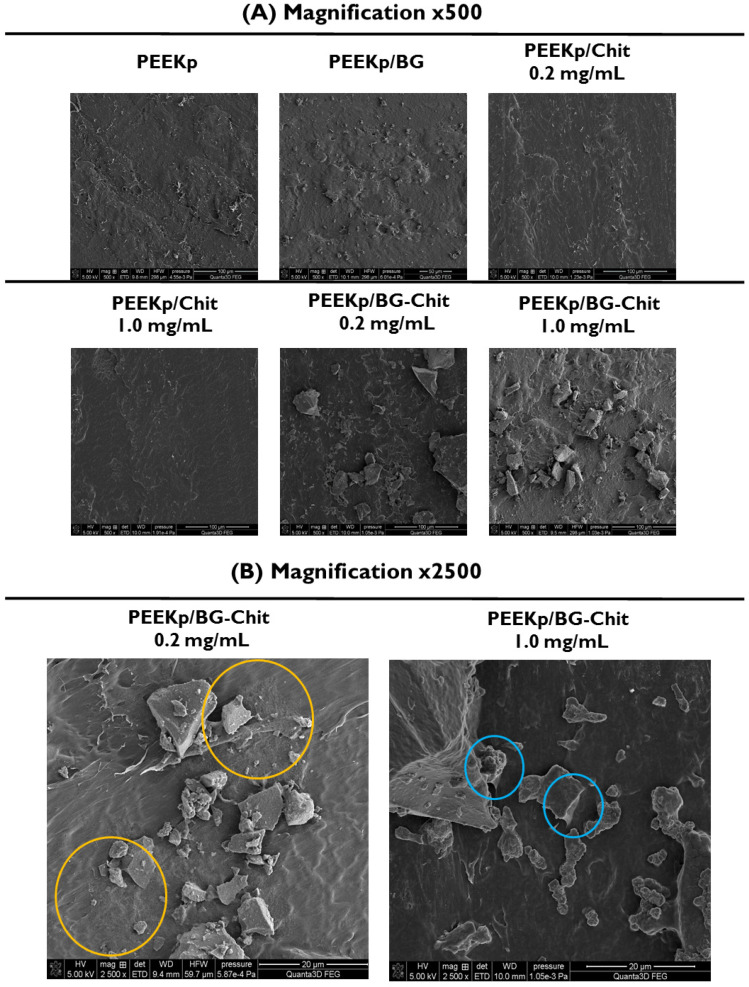
(**A**). SEM images of the modified PEEK surfaces with ×500 magnitude. The yellow circles show the structure of chitosan films. (**B**). SEM images of PEEKp/BG-Chit 0.2 and 1.0 mg/mL with ×2500 magnitude for better visualization of the BG−Chit mixed coatings. The blue circles denote bioglass fusion sites with the solid support.

Scanning electron microscopy was used to evaluate the structures obtained during the modification of the PEEK surface (Figure 2A). The first crystal-like structures were observed after the adhesion of bioglass to the activated PEEK. These structures were scattered regularly and were characterized by irregular shapes. Their sizes were different, but they did not exceed the size of 10 × 10 μm^2^. This constancy can be the effect of the concentration of polar functional groups on the PEEK surface, which supports the adhesion of bioglass to its surface. When the chitosan layer was present on the activated PEEK, no specific structures were observed, but rather the presence of a smoothing layer compared to the PEEKp control sample (Figure 2A). Considering the experiments by the other authors, where the chitosan layers on PEEK substrate were investigated, the structures varied depending on the deposition technique, chitosan molecular properties, and concentration, as well as prefabrication of the PEEK surface [31,32].

Finally, for both BG-Chit mixed coatings deposited on the PEEKp substrate, significantly larger crystal structures of bioglass compared with the PEEKp/BG were observed (Figure 2A). Additionally, on the crystals surface, the presence of an additional lattice-like coating was noticed (in particular, for the chitosan concentration of 1mg/mL) connecting the crystals with the surface of the PEEK polymer. At this point, the structure of chitosan appeared and, for the better visualization of the BG-Chit mixed coating, larger magnitude images (×2500) were presented in Figure 2B. For the lower concentration of chitosan (0.2 mg/mL), the presence of chitosan structures was observed on the bioglass crystallites and near them (marked in yellow), thus the PEEK surface was partially covered with chitosan, or the chitosan molecules accumulated to form visible aggregates. When the BG- Chit mixed coating was deposited from the larger concentration of chitosan (1.0 mg/mL), the chitosan-like structure was present on both BG and PEEK materials, making the coating more homogeneous. Similar results were described by Ravarian et al., when after drying the chitosan-bioglass sol-gel hybrids, they observed rigid monolith surfaces with visible bioglass crystals [33]. The other studies based on the EPD technique to produce the BG-Chit coating on the modified PEEK surfaces reported a more homogeneous coating layer but still with clearly visible BG crystals, very well bound to the substrate [17]. In our case, probable binding with the surface was observed at the point of contact between the crystallites and the PEEK surface (marked with blue circles). This can suggest that at different concentrations chitosan with the bioglass adhere to the activated PEEK surface differently, revealing inconsistent structures and coating properties. However, Hong et al. observed insufficient BG-Chit adhesion to the sandblasted PEEK surface, which prevented the preparation of the samples [6]. Thus, the sample wettability and/or energetic properties of PEEK are necessary to obtain a robust adhesion of the BG-Chit coatings. In our experiment, this was successfully achieved by the plasma activation of the PEEK polymer.

### 2.3. TOF-SIMS Analysis

**Figure 3:**
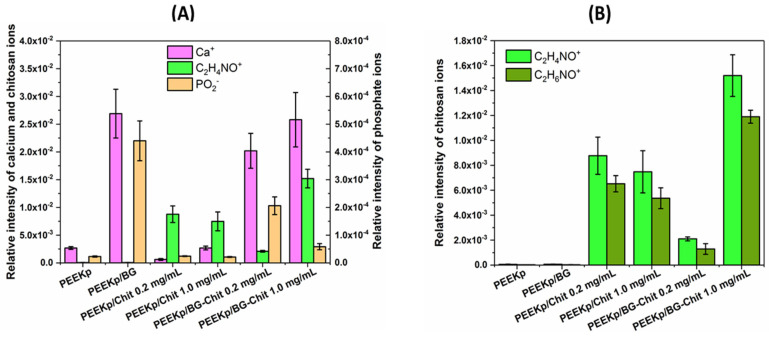
(**A**). Relative intensities of calcium ($Ca^{+}$), phosphate ($PO_{2}^{-}$), and chitosan ($C_{2}H_{4}NO^{+}$) ions determined on the modified PEEK surfaces. (**B**). Relative intensities of chitosan ($C_{2}H_{4}NO^{+}$ and $C_{2}H_{6}NO^{+}$) characteristic ions determined on the modified PEEK surfaces.

The secondary ion mass spectrometry coupled with the time-of-flight detector is a novel technique allowing for the determination of chemical composition of solids, and thus was exploited to develop the chemical composition of the modified PEEK surfaces. Figure 3A depicts the intensity distribution of the calcium, chitosan, and phosphate ions for the multi-step modification of the PEEK substrate. In the TOF-SIMS spectra, the signals of $Ca^{+}$ and $PO_{2}^{-}$ fragments are typical artifact peaks identified for most substrates that are not covered with a specific layer or very thin layers. Therefore, small artifact signals were observed for the PEEKp and PEEKp/Chit surfaces (Figure 3A).

The largest abundance of $Ca^{+}$ fragment is observed for the layers with the bioglass, which proves its presence on the modified PEEK. Slightly larger intensities of calcium were observed for BG-Chit 1.0 mg/mL, compared to 0.2 mg/mL. This can be related to the enhancement of the excitation efficiency of calcium ions in the presence of larger amounts of chitosan and by the different distribution of the $Ca^{2+}$ ions within the BG-Chit layer. At the lower concentration of chitosan, the calcium ions $Ca^{2+}$ reside in closer proximity to each other, whilst at higher chitosan concentration, the calcium ions are more isolated. On the other hand, the distribution of intensity of $PO_{2}^{-}$ is proportional to the amount of bioglass within the BG-Chit layer. The phosphate groups are incorporated into the $SiO_{2}$ network by chemical bonds (one phosphorous atom is connected to two oxygen atoms), while calcium ions are trapped by interactions with the hydroxyl groups [34]. Some $Ca^{+}$ ions were observed for the chitosan layers deposited on the PEEK without the bioglass, as well as for the PEEKp substrate, however, this was a result of the artifact signals. For the chitosan identification, two of the most prominent chitosan fragments, $C_{2}H_{4}NO^{+}$ and $C_{2}H_{6}NO^{+}$, were identified for all chitosan-containing layers (Figure 3B).

These ions were already described for the chitosan identification in our previous papers [11,35] and were found to be complementary; thus, we decided to use the $C_{2}H_{4}NO^{+}$ ion for the chitosan description (Figure 3A). The slightly smaller intensity of the $C_{2}H_{4}NO^{+}$ chitosan fragment is identified for the layer deposited from the more concentrated solution (PEEKp/Chit 1.0 mg/mL). In this case, the chitosan layer is thicker, thus the yield of the chitosan fragment received from the denser and thicker layer is diminished. This is opposite to the observed yield of the phospholipid monolayers, examined using the TOF- SIMS analyser, where the intensity of secondary ions is proportional to the concentration of the components in the monolayer [35,36]. However, the intensity of the chitosan fragments is roughly proportional to the concentration of chitosan in the BG-Chit layer. This is very likely determined by the chitosan molecular rearrangement within this layer. The chitosan molecules might not be cross-linked as in the pure chitosan layer due to the bioglass environment. Consequently, the intensity of the chitosan fragment in the BG-Chit coating reflects the layer composition. On the other hand, the SEM images revealed a specific location of chitosan when co-adsorbed with the bioglass (Figure 2A,B). Thus, at the low concentration of chitosan when both components attach to the polymer surface, the chitosan can remain between the PEEK and the bioglass surface connecting each other. In this case, more bioglass-related ions and fewer chitosan ions would appear. The effect would be different when more chitosan is present. Therefore, chitosan could create more cross-linked structures and cover more bioglass, thus less bioglass ions and more chitosan ions will appear (Figure 3A).

**Figure 4:**
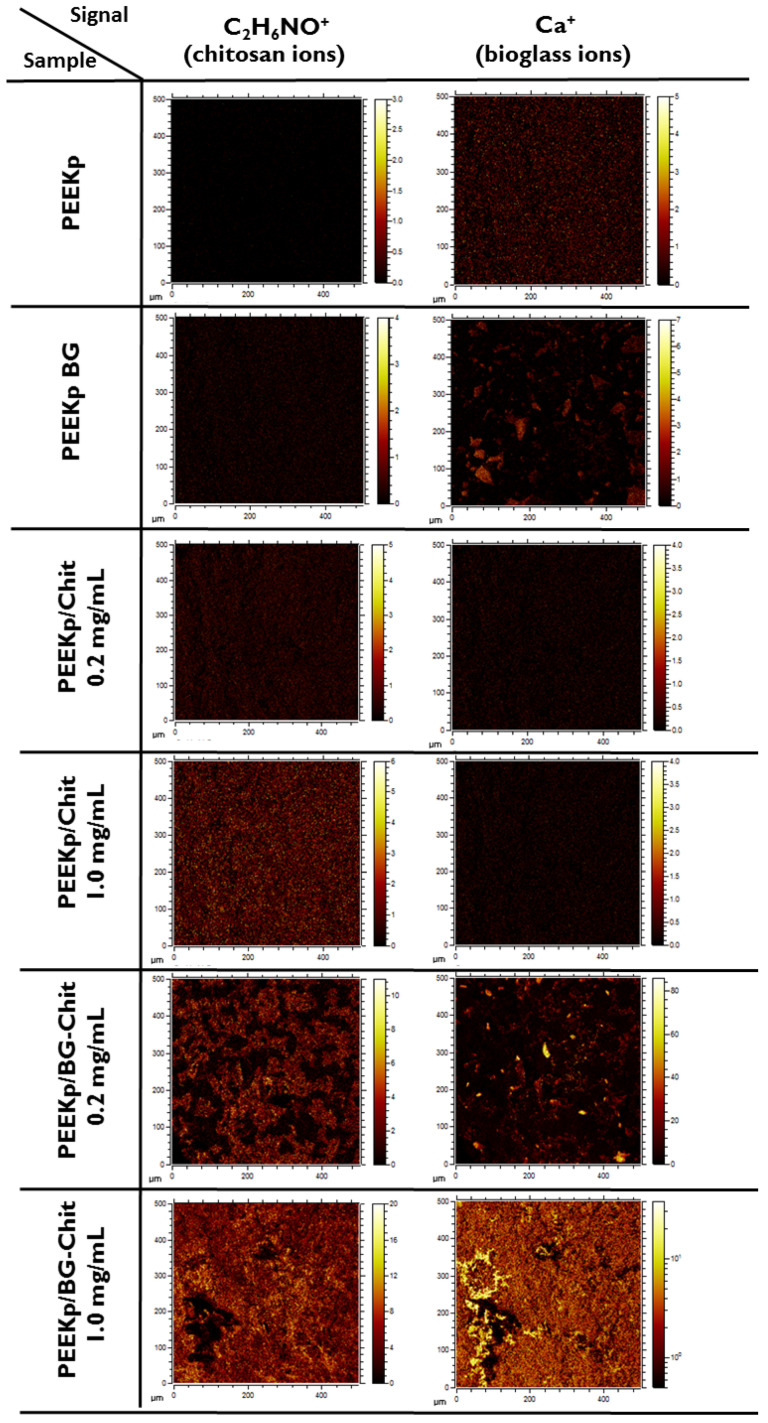
Lateral distribution of chitosan ($C_{2}H_{4}NO^{+}$) and calcium ions $\left(Ca^{+}\right)$ obtained for the modified PEEK surfaces.

Figure 4 presents the chemical maps of chitosan and calcium ions for the investigated PEEK samples. Their intensities are in agreement with the quantitative analysis (Figure 3A) and reveal specific bioglass and bioglass-chitosan structures and distribution (Figure 4). 

**Figure 5:**
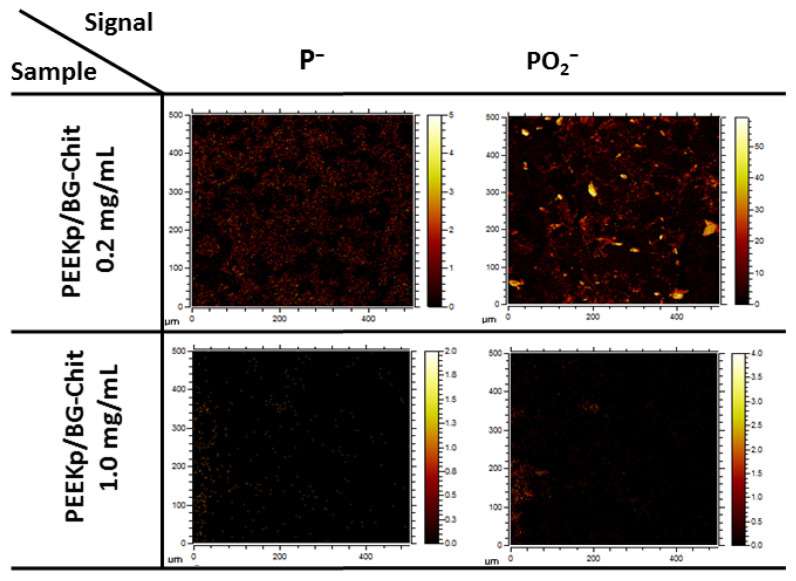
Lateral distribution of phosphorus $P^{-}$ and phosphate $PO_{2}^{-}$ ions for PEEKp/BG-Chit 0.2 and 1.0 mg/mL mixed coatings.

It was particularly visible for the PEEKp/BG-Chit 0.2 mg/mL, where the chitosan ions only partially covered the modified surface, while for the larger chitosan concentration, the chitosan coverage looked more homogeneous. The chemical maps for the calcium ions revealed bioglass related structures, notably evident for the PEEKp/BG and PEEKp/BG-Chit 0.2 mg/mL surfaces (Figure 4). Since the calcium ion intensities and distribution for PEEKp/BG-Chit 1.0 mg/mL were unusual, we compared the $P^{-}$ and $PO_{2}^{-}$ chemical maps for both BG-Chit mixed coatings (Figure 5).

**Figure 6:**
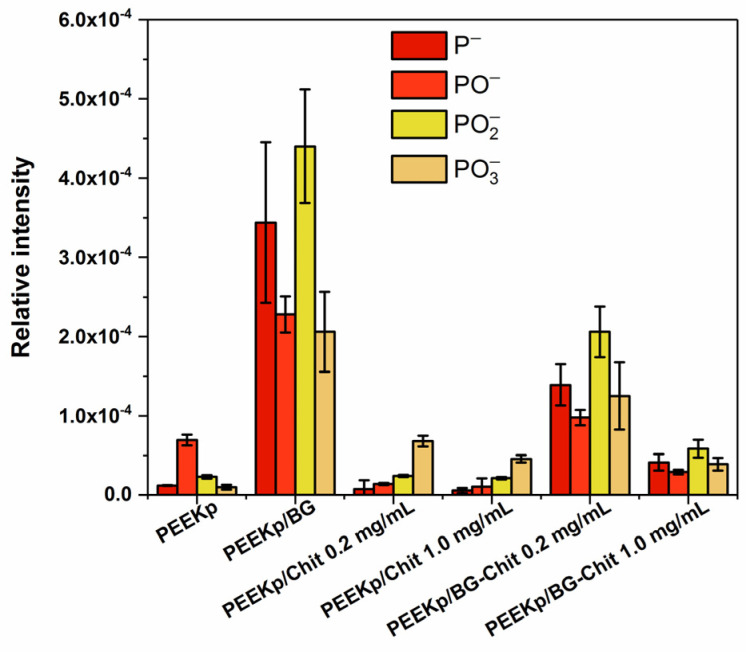
Relative intensities of phosphorous $\left(P^{-}\right)$ and phosphate $(PO^{-}$,$\;PO_{2}^{-}$, $PO_{3}^{-})$ moieties determined on the modified PEEK surfaces.

The results were consistent with the ion distribution presented in Figure 3 and Figure 4. Thus, there could be a certain mechanism that enhances the calcium ionization and distribution for higher concentrations of chitosan in the BG-Chit hybrid coatings obtained on the PEEKp surfaces. Apart from the $PO_{2}^{-}$ fragment that was shown in Figure 3A, phosphorous $\left(P^{-}\right)$ and phosphate ions $(PO^{-}$, $PO_{3}^{-})$, corresponding to the bioglass were identified (Figure 6).

The distribution of all phosphate fragments in the bioglass-containing samples was very similar. Nevertheless, the differences of their intensities were significant. Again, this can be correlated with the presence of chitosan on the BG crystals (or the opposite effect), and the specific concentration/grouping process of the BG-Chit suspension on the PEEK surfaces. On the other hand, bioglass can penetrate the chitosan structures and the unique swelling properties of chitosan can have an additional effect for the greater concentrations of chitosan that are present in the coating.

### 2.4. Wettability and Surface Free Energy of the Modified PEEK Surfaces

**Figure 7:**
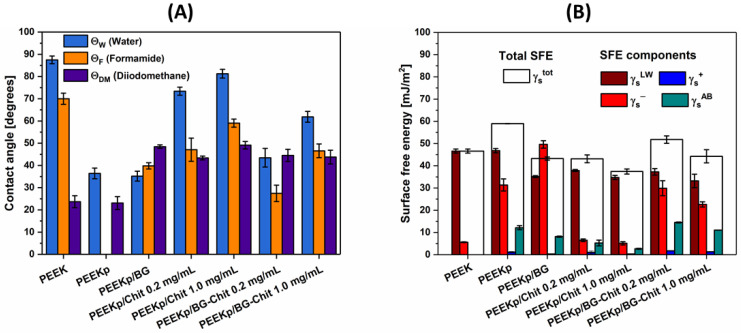
(**A**). Mean contact angle of water, formamide, and diiodomethane, and (**B**) surface free energy, as well as its components determined for the modified PEEK surfaces. The error bars in (**A**) denote standard deviations from 10–15 contact angle values of single test liquid of each surface (twice). The error bars in (**B**) denote the maximum and minimum values of surface free energy and its components calculated for 2 samples of each studied surface from the 10–15 contact angle values of each liquid (their averages).

Wetting properties of solids are one of the key surface parameters which have a significant effect on the host response at the cellular level. This phenomenon is mostly associated with the adsorption of the proteins and their conformation. Based on numerous experiments, the scientists established that the optimal water contact angle which best induces the cell proliferation, attachment, and spreading process occurs between 55.0–70.0° [37,38]. Figure 7A presents the contact angles of two polar (water, formamide) and one nonpolar (diiodomethane) test liquids, measured on differently modified PEEK surfaces.

The commercial PEEK revealed a hydrophobic character (contact angle of water $\Theta_{W\;}=$ 87.5°), which is consistent with the findings of other authors and our previous studies [39,40]. The contact angle for more polar formamide was estimated to be $\Theta_{F}=$ 70.0° and $\Theta_{D}=$ 23.7° for the non-polar diiodomethane. The hydrophilic-hydrophobic character of the PEEK surface changed significantly after the plasma treatment ($\Theta_{W\;}=$ 36.4°). This was due to the surface activation and creation of multiple polar groups on the PEEK surface rich in oxygen and nitrogen. The observed effect was even larger for formamide ($\Theta_{F}=$ 0.0°—total wetting), but not noticeable in the case of diiodomethane ($\Theta_{D}=$ 23.1°).

The contact angles of water did not change significantly after the deposition of BG ($\Theta_{W\;}=$ 35.2°), nevertheless, it was possible to measure the contact angle of formamide ($\Theta_{F}=$ 39.8°) (Figure 7A). Additionally, the contact angle of diiodomethane changed significantly and was equal to $\Theta_{D}=$ 48.5°. Considering the SEM images, the activated PEEK surface was found to be covered evenly but partially with the BG. Those contact angles were the result of the wettability of the activated PEEK surface and bioglass. When the plasma-activated PEEK surfaces were covered with the chitosan layers obtained from two different chitosan concentrations (0.2 mg/mL or 1.0 mg/mL), the contact angles of water and formamide decreased compared to the PEEK control. For 0.2 mg/mL, the contact angle of water was $\Theta_{W\;}=$ 73.4°, while that of formamide was equal to $\Theta_{F}=$ 47.0° (Figure 7A). This observation can suggest that chitosan binds to the highly polar (activated) PEEK surface via its polar groups (–OH; –NH_2_), and orients its non-polar residues towards the air phase. In the case of concentration of chitosan that is five-times greater (1.0 mg/mL), the PEEK surface was less polar, revealing contact angles of water and formamide equal to $\Theta_{W\;}=$ 81.3° and $\Theta_{F}=$ 59.0°. Since the polar character of PEEK covered with chitosan decreased at a greater concentration of chitosan, it can be concluded that the coverage of PEEK increased, and the probe liquids had less space to contact/interact with the activated substrate.

The most interesting observations were made when the BG-Chit mixed coatings were deposited on the activated PEEK surface. For the smaller chitosan concentration (0.2 mg/mL), significant decreases in the water ($\Theta_{W\;}=$ 43.5°) and formamide ($\Theta_{F}=$ 27.4°) contact angles were observed (Figure 7A). This was probably due to the preferential binding of chitosan with the bioglass crystallites. Thus, the chitosan can cover the PEEK surfaces only slightly and remain concentrated on the BG crystallites. This can result in a significant increase in the accessibility of the polar PEEK surface to the test liquids. For the latter examined system with the largest amount of chitosan, an increase in water ($\Theta_{W\;}=$ 61.9°) and formamide ($\Theta_{F}=$ 46.5°) contact angles compared with the system with a smaller chitosan concentration was found. This phenomenon can also be explained by the preferential adsorption of chitosan on the BG crystallites, followed by adsorption on the PEEK surface. Nevertheless, at the higher concentration of chitosan with bioglass, the separation of the BG-Chit suspension in the solution was observed. Hence, a creation of something that resembles the bonds between chitosan and bioglass and their specific adsorption on the activated PEEK surface cannot be ruled-out. Considering the optimal wettability for the biocompatibility assessment ($\Theta_{W\;}=$ 55.0–70.0°), the PEEKp/BG-Chit 1 mg/mL system achieved the best value of water contact angle ($\Theta_{W\;}=$ 61.9°) [38]. The other studies investigating the BG-Chit materials in terms of their wetting properties reported water contact angle values of 45.0–55.0°, which were also considered to be suitable for osteoblast cell adhesion [17,41].

The measurements of contact angles with a wide range of well characterized probe liquids on the solid surfaces are the most common method for the determination of the surface free energy (SFE) and its components. Generally, SFE is the rise in energy strictly connected with moving the atom of the material from its bulk to its surface. When the new surface is created, these atoms reveal coordinative unsaturation of the bonds because now they are exposed to the different environment (air phase) [42]. Hence, the new surface atoms are characterized by different energies compared to the bulk atoms and SFE is the measure of this difference in the type and number of outer bonds. Primarily, when these bonds are mostly Lifshitz-van der Waals in type, SFE will exhibit non-polar nature. On the other hand, when the outer bonds are mostly the ionic/covalent type, there will be a greater contribution of the Lewis acid-base to the total SFE of material.

PEEK itself exhibits relatively low values of total SFE ($\gamma_{s}^{tot}=$ 46.6 mJ/m^2^) and mainly consists of the non-polar Lifshitz-van der Waals ($\gamma_{s}^{LW}$) component and minor electron-donor ($\gamma_{s}^{-}$) parameter (Figure 7B). The lack of the Lewis acid-base component ($\gamma_{s}^{AB}$) can suggest why PEEK is strongly inert to the biological environment. The cold air plasma treatment of the PEEK surface affects $\gamma_{s}^{tot}$ largely, and particularly $\gamma_{s}^{-}$ (Figure 7B). Moreover, this step causes the appearance of the $\gamma_{s}^{AB}$ component, which is equal to 12.1 mJ/m^2^. Unfortunately, this effect weakens over time and the PEEK surface returns to its original state in about 10 days. Similar observations were described by the other authors when the PEEK substrate was treated with cold oxygen plasma [40]. The adsorption of the bioglass crystallites on the plasma activated PEEK surfaces resulted in a significant increase in the electron-donor parameter ($\gamma_{s}^{-}$) compared with the PEEK control and the plasma activated PEEK (Figure 7B). This was undoubtedly the result of the contact of the measuring liquids with the bioglass structures rich in oxygen and silicon, as well as sodium and calcium ions. When the chitosan layers from the two different solution concentrations were adsorbed on the activated PEEK surface, its ionic nature of bonds was clearly suppressed as a result of blocking the polar groups by the chitosan layer. The more chitosan, the more suppressive effect was observed, however, only in the value of the total surface free energy $\gamma_{s}^{tot}=$ 43.1 and 37.4 mJ/m^2^ for PEEKp/Chit 0.2 mg/mL and PEEKp/Chit 1.0 mg/mL, respectively. Nevertheless, for both chitosan coatings, the Lewis acid-base component remained and was determined at the similar level (Figure 7B).

For the mixed BG-Chit layers deposited on the activated PEEK surface, relatively high values of the surface free energy were observed, which were different depending on the amount of chitosan. At the smaller ratio of chitosan (0.2 mg/mL) $\gamma_{s}^{tot}$ increased to 51.8 mJ/m^2^ and the highest value of $\gamma_{s}^{AB}$ of 14.5 mJ/m^2^ was found. Simultaneously, this surface revealed the electron-donor ($\gamma_{s}^{-}=$ 29.9 mJ/m^2^) and electron-acceptor ($\gamma_{s}^{+}=$ 1.76 mJ/m^2^) parameters which were close to the value obtained for the plasma activated PEEK ($\gamma_{s}^{-}=$ 31.4 mJ/m^2^, $\gamma_{s}^{+}=$ 1.17 mJ/m^2^). For the twin sample with a larger chitosan content (1.0 mg/mL), the obtained parameters of the surface free energy were very close, but $\gamma_{s}^{-}$ decreased slightly and, in consequence, reduced $\gamma_{s}^{tot}$ by 7.5 mJ/m^2^ (Figure 7B). The described values point to the formation of cation and anion sites from the chitosan and bioglass moieties on the PEEK surface modified by the BG-Chit coating.

**Figure 8:**
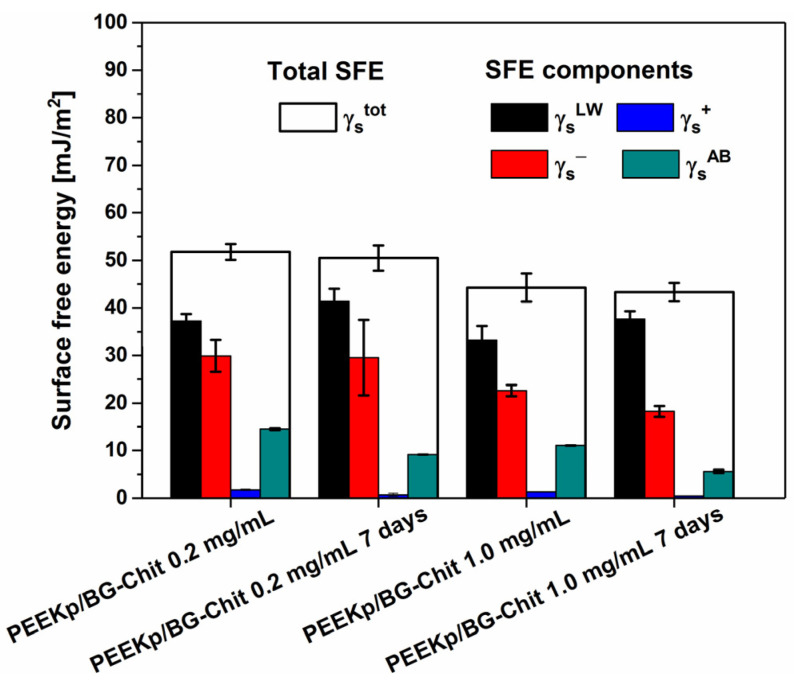
Surface free energy and its components, determined for modified PEEK surfaces, fresh and after 7 days. The error bars denote the maximum and minimum values of surface free energy and its components calculated for 2 samples of each studied surface from the 10–15 contact angle values of each liquid (their averages).

Additionally, for PEEKp/BG-Chit 0.2 and 1.0 mg/mL, the energetic surface parameters were determined after 7 days and no significant differences in the surface free energy parameters were found (Figure 8).

At this point, this is a very promising observation because for the long lasting charged surface sites, it is possible for the different molecules (from ions to proteins) to interact with the surface through the ion-dipole attractions and orbital overlap. Considering the complexity of the tissue regeneration process in this stage, it is important that specific modifications introduce permanent and pre-planned changes regarding the chemistry and surface properties of the potential bone replacement materials.

### 2.5. Biocompatibility Assessment Applying Incubation in the Simulated Body Fluid

The in vitro bioactivity studies include research based on biomaterial incubation in the simulated body fluid (SBF). The SBF solution contains all the necessary inorganic components of human blood plasma. The incubation of materials is made at a temperature close to the physiological temperature of the human body (37 °C). The pH value of the SBF solution (7.25 or 7.40) is also close to the physiological pH value of human blood (7.45). After the sample incubation in the artificial blood plasma, changes of its surface morphology and surface chemistry take place. Indicating the formation of the phosphate-calcium forms related to the material bioactivity, it is possible to predict and estimate the material usefulness. In optimal conditions when the new structures appear, the examined surface is covered with cauliflower-like forms of calcium phosphates. The resulting material, in terms of morphology, is characteristic ofcrystalline hydroxyapatite (HAP) ${\left(Ca_{10}PO_{4}\right)}_{6}{\left(OH\right)}_{2}$ [43].

**Figure 9:**
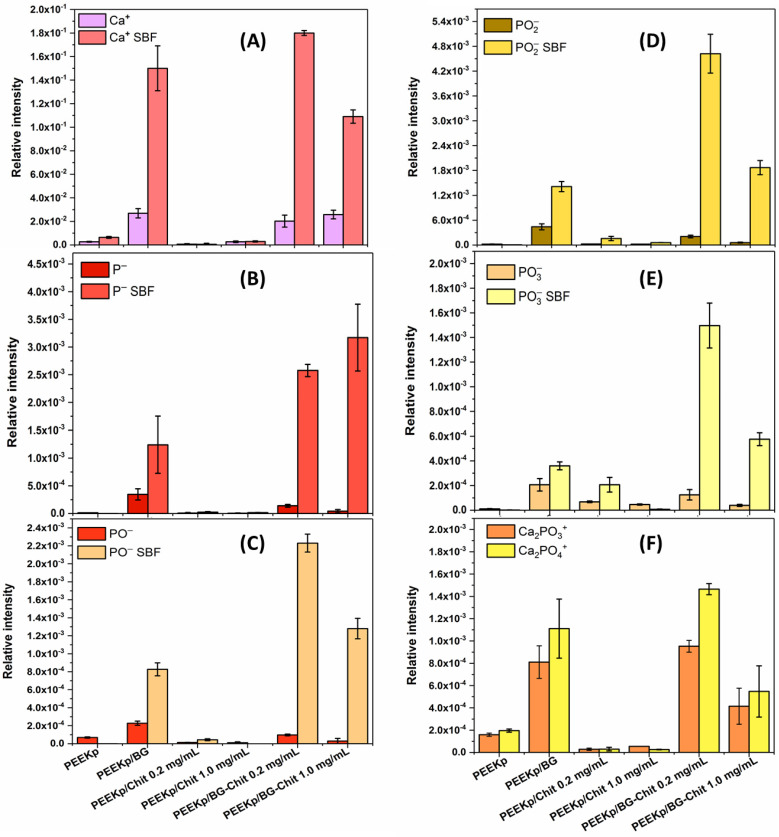
(**A**) Relative intensities of calcium $(Ca^{+}$), (**B**) phosphorus ($P^{-}$), phosphate fragments (**C**) $PO^{-}$, (**D**) $PO_{2}^{-}$, and (**E**) $PO_{3}^{-}$ ions determined on the modified PEEK surfaces before and after the incubation in SBF. Figure (**F**) calcium phosphate $Ca_{2}PO_{3}^{+}$ and $Ca_{2}PO_{4}^{+}$ ions appeared after incubation process.

In our experiment, after the incubation in SBF, a strong intensity increase in the $Ca^{+}$ fragment is observed for the BG-containing samples (Figure 9A). For the chitosan-containing PEEKp, no difference is visible, while the activated PEEK polymer substrate demonstrated only a slight increase in the $Ca^{+}$ amount. The additional information about the hydroxyapatite layer is provided by the $P^{-},\;PO^{-},\;PO_{2}^{-},PO_{3}^{-}$ fragments (Figure 9B–E). There is a similar correlation between the different types of ions identified by TOF-SIMS and the formation of hydroxyapatite. They exhibit significantly greater intensities for the BG-Chit mixed layers compared with the single BG or the chitosan layers. However, greater intensities were always determined for the smaller chitosan ratio (PEEKp/BG- Chit 0.2 mg/mL), except $P^{-}$ ions, which are single atoms and, thus, cannot reflect the current structure sufficiently. Finally, the new ions, $Ca_{2}PO_{3}^{+}$ and $Ca_{2}PO_{4}^{+}$, were identified (Figure 9F). These ions were already established as corresponding to the HAP structures in the studies applying the TOF-SIMS technique [44]. Their intensities were larger and similar for the pure BG and BG-Chit 0.2 mg/mL and half smaller compared with BG-Chit 1.0 mg/mL. Moreover, the lack of these signals was observed for the PEEK/Chit 0.2 and 1.0 mg/mL samples, while for PEEKp control sample, the noticeable presence of calcium phosphate ions with their proper correlation was observed (Figure 9F). This suggests that the HAP formation on the PEEK surface was suppressed by the presence of chitosan. Similar results were obtained by Fraga et al. when the combination of the sodium silicate with chitosan allowed obtaining the HAP on its surface during the incubation in SBF [45]. Nevertheless, in our experiment this can be associated with blocking the most active PEEKp and bioglass sites by chitosan and, thus, affecting the HAP formation. Moreover, for the PEEKp/BG-Chit 1.0 mg/mL, the smaller intensity ratios of $Ca_{2}PO_{3}^{+}$ and $Ca_{2}PO_{4}^{+}$ suggest a slightly different molecular arrangement of the hydroxyapatite molecules on the surface, compared with the smaller amount of chitosan. The intensity distribution of the $PO^{-},\;\;PO_{2}^{-}$, $PO_{3}^{-}$ fragments for BG-Chit 1.0 mg/mL and BG-Chit 0.2 mg/mL correlates properly with the distribution of $Ca_{2}PO_{4}^{+}$, which exhibits a greater amount of HAP for BG-Chit 0.2 mg/mL. Surprisingly, the intensity of the negative phosphate ions corresponding to the HAP deposited for the pure bioglass (PEEKp/BG) was significantly smaller than that observed for BG/Chit 0.2 mg/mL and BG/Chit 1.0 mg/mL.

**Figure 10:**
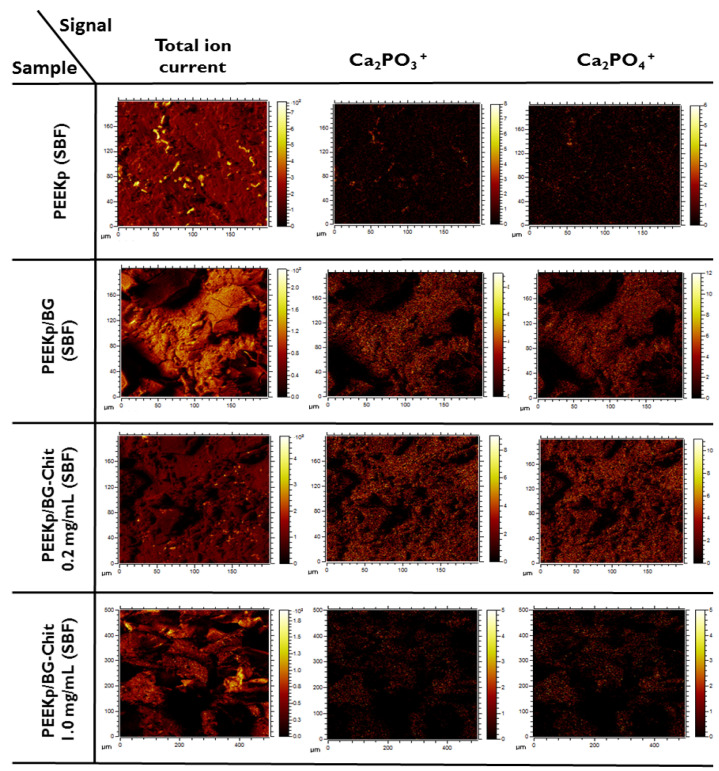
Total ion current and lateral distribution of calcium phosphates $(Ca_{2}PO_{3}^{+}$ and $Ca_{2}PO_{4}^{+})$ ions corresponding to hydroxyapatite.

This can be determined by the different yield of the negative ions from hydroxyapatite bound on the pure bioglass. This suggests that the chemical bonds and molecular arrangements of HAP layer on the single bioglass are different compared with the HAP deposited on bioglass-chitosan coating. It can be hypothesized that, in such systems, it is important to select the optimal concentration of chitosan. Too high a concentration of this polysaccharide can lead to a strong swelling process and, thus, block the formation of hydroxyapatite on the bioglass crystallites. The additional insight into these questions can be provided by the chemical maps that show the distributions of individual components within the layer with the lateral resolution below 0.5 µm (Figure 10).

**Figure 11:**
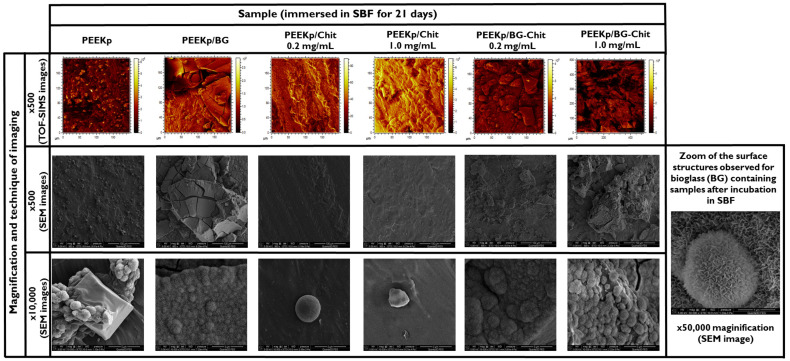
TOF-SIMS and SEM images of the modified PEEK surfaces after the immersion in SBF for 21 days.

The distribution of the most prominent hydroxyapatite $Ca_{2}PO_{3}^{+}$ and $Ca_{2}PO_{4}^{+}$ fragments is presented in Figure 10. On the PEEKp substrate only, several HAP related island-like areas are visible that correspond to the high intensity points in the total ion current image (Figure 9). For the pure bioglass, larger crystalline structures were observed. After mixing BG with chitosan (PEEKp/BG-Chit 0.2 mg/mL), a fine-crystalline structure of HAP layer was present. When the concentration of chitosan increased (PEEKp/BG-Chit 1.0 mg/mL), significantly bigger crystals were observed. Simultaneously, the absolute value of HAP decreased notably (about 10-times) together with the total ion current (3-times). In consequence, the relative intensity decreases about 3-times that corresponds to the distribution shown in Figure 9F. Figure 11 depicts the SIMS and SEM images of the modified PEEK structures after incubation in SBF.

After the incubation in the SBF process, all of the investigated PEEK surfaces revealed the creation of new structures. On the plasma activated PEEK, small (max. 20 × 20 μm^2^) apatite-like structures were observed as the small islands scattered over the whole surface. Interestingly, the cauliflower structures grew not directly from PEEK surface, but from the ice crystal-like structures created on it (Figure 11). This can be related to the most active PEEK sites after the plasma activation ($-CO,-COO,-CNH_{x},-CN<)$, which induced the crystallization and mineralization of new structures. On the other hand, for the pure bioglass deposited on the PEEKp polymer, very intense crystal growth was observed, and the new HAP layer mineralized not only on the BG crystals, but also on the PEEKp surface. Additionally, the structures visible on both the SIMS and SEM images are partially cracked. At the larger magnitude (×10,000), the coverage revealed sponge-like mineral structures (Figure 11). The situation was different for the surfaces that contained only chitosan. No HAP-related structures were present. Only small spherical crystals (2.5 μm diameter) were unevenly scattered on both the PEEKp/Chit 0.2 and 1.0 mg/mL surfaces. It can be hypothesized that the chitosan deposited onto the plasma activated PEEK surface inhibits the formation of apatite-like structures. For the BG-Chit mixed coatings, fine crystals covered with newly crystallized structures were observed. In this case, no cracked sites were present. This can be a result of the incorporation of the chitosan to its structure and enhancing its flexibility. Considering the micro-structure of the obtained surfaces, it can be concluded that at higher concentration of chitosan (1.0 mg/mL), less homogenous structures appeared, and more isolated islands were present compared with the smaller concentration of chitosan (0.2 mg/mL) (Figure 11). Our results are in agreement with the other studies where the presence of bioglass in the chitosan coating always enhanced the formation of the porous apatite structures [16,18]. These structures appeared even after 3 days in SBF and were less porous with the more time spent in SBF. However, in our experiment new apatite structures maintained their porous structure after 21 days of immersing in SBF.

**Figure 12:**
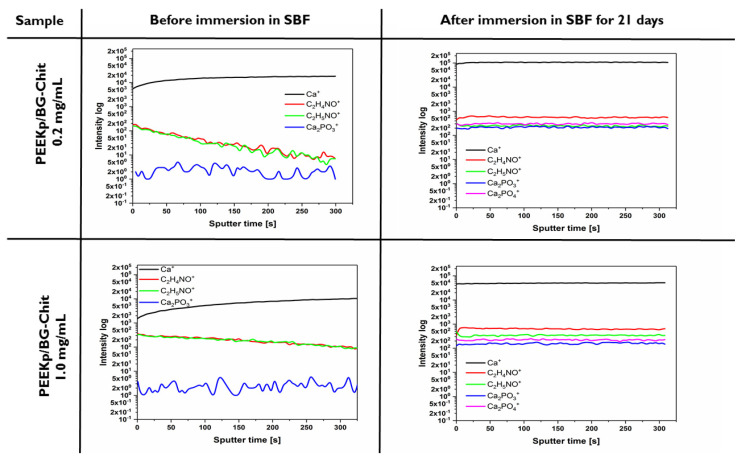
Depth profiles determined before and after the incubation in SBF for PEEKp/BG-Chit 0.2 and 1.0 mg/mL systems.

For the in-depth structure analysis of newly obtained surfaces, the distribution of chitosan and HAP related ions was performed applying depth profiling (Figure 12).

Before the incubation in SBF, no HAP related signals ($Ca_{2}PO_{3}^{+}$ and $Ca_{2}PO_{4}^{+}$) were observed. On the other hand, the intensity of the $\;Ca^{+}$ ions increased as a function of the sputtering time (depth of sample), while the intensity of chitosan ions decreased. A similar effect was observed for both BG-Chit mixed layers deposited on the PEEKp surface. This confirms the hypothesis that chitosan is adsorbed on the surface of the bioglass crystallites and remains in the topmost layer of the coating. A significantly different behavior was observed for the PEEK/BG-Chit samples after the incubation. All ions related to the bioglass, chitosan, and hydroxyapatite demonstrated constant intensity as a function of the sputtering time (Figure 12). This could indicate the homogeneous distribution of each individual component as a function of depth of the investigated samples. The HAP formation during the incubation in SBF is a complex process and involves a few steps containing $K^{+}$ and $Na^{+}$ exchanging with $H^{+}$ or $H_{3}O^{+}$; the formation of silica layer and then the breaking of O−Si−O connections, condensation, and polymerization of silanol groups on the surface of BG; the deposition of $Ca^{2+}$ ions; and finally the crystallization of hydroxyapatite. In the investigated systems when the whole process is induced by the presence of bioglass on the PEEKp surface, the chitosan molecules can possibly migrate from the coating and create a new forming structure. However, the other studies reported the degradation of chitosan when the BG-Chit-lawsone hybrid coatings formed by the EPD technique were immersed in SBF [17]. This discrepancy can be a result of different coating and/or different substrate preparation procedure. During this process, positively charged chitosan molecules in suspension interacted with the hydroxyl groups on the bioactive glass particles surface to form hydrogen bonds, which leads to the co-adsorption of chitosan and glass on the support [46].

The described experiments are very important from the point of view of designing biomaterials with specific properties. For example, tests of wettability connected with the surface free energy estimation allow the selection of the optimal surface in terms of its energy for the best cell adhesion and proliferation. This goes along with the surface topography which can be examined by different imaging techniques (e.g., SEM) to ensure the most appropriate conditions for cell attachment. Moreover, the surface chemistry measurement techniques, such as FTIR, XRD, and TOF-SIMS, are usually applied for the coatings investigations to establish their composition, structure, stability, or the presence of additional bioactive substances, e.g., drugs. Finally, the incubation tests in SBF provide very useful information about if and how fast apatite structure appears on the material, which helps predict its biocompatibility.

From the implantology point of view, the presented material (PEEK) shows the beneficial properties such as: mechanical properties (modulus of elasticity and tensile as well as yield strength, hardness, and toughness), which are very similar to those of human bone, corrosion resistivity, and bioinertness [47]. Due to this, it was already introduced for application as the spinal cage, dental materials, knee joints, and many other bone replacements [3,48,49]. The enrichment of PEEK with the bioglass-chitosan coating improved its surface properties, mainly wettability and roughness, which now are in the more optimal range for osteoblasts adhesion (water contact angle of 55.0–70.0° and roughness 1–3 μm) [37,38]. Nevertheless, the proposed material is not without its limitations. Firstly, it can be considered as unknown for the long-term stability of the coating. Secondly, it is associated with the high geometry of dental implants, so more studies are required to examine plasma activation and film deposition for such materials (bone screws). Finally, a serious limitation occurs with the implant placement. However, the degradation of the coating during the strong friction associated with the installation of the implant in the osteon or the trabeculae of the bone should be ruled out. In fact, for the spinal cages implants there would be beneficial fusion of two vertebrae with a bone, unless there is a pressure on the spinal nerves and the spinal cord itself. The PEEK based implants would fit better where the metal ones generate mechanical overstress (spinal cages, knee joints). Moreover, due to the 3D printing technology, they can be superior to the metallic implants where complicated shapes are required (tooth screws, skulls, and face bones implants). Finally, PEEK does not induce allergy, compared to the metal alloys; thus, it can be in contact with a large surface area of living tissue and also its color is more appropriate, taking the esthetic implants into account.

## 3. Materials and Methods

### 3.1. Materials

The 20 × 30 × 5 mm^3^ PEEK plates were cut from the commercially available TECAPEEK natural (1000 × 500 × 5 mm^3^, PROFILEX) and were used as a substrate. The chitosan (molecular weight of 100,000–300,000 and the deacetylation degree (DD) of 82.0–84.0%) was purchased from Acrōs Organics (Geel, Belgium). The bioglass (45S5) was obtained from XL Sci-Tech, Inc. (Richland, WA, USA) in the form of granules (45–300 μm size, IsoSpheres, Richland, WA, USA). Before use, the bioglass powder was additionally ground in an agate mortar. Both materials were applied for PEEK modification. Methanol, acetic, and hydrochloric acid were purchased from Avantor Performance Materials Poland S.A. with a purity above 99.0%. Each time, the Milli-Q system water (resistivity ~18.2 MΩ*cm) was used. For the preparation of the simulated body fluid (SBF), a set of the following chemicals was used: NaCl, ${\mathrm{Na}}_{2}{\mathrm{CO}}_{3}$, ${\mathrm{Na}}_{2}{\mathrm{HPO}}_{4}\times 12{\;H}_{2}O$, ${\mathrm{MgCl}}_{2}\times 6{\;H}_{2}O$, and ${\mathrm{CaCl}}_{2}$ obtained from POCh (Gliwice, Poland); KCl, ${\mathrm{Na}}_{2}{\mathrm{SO}}_{4}$ from Chempur (Piekary Śląskie, Poland); and Tris(hydroxymethyl)aminomethane (${\mathrm{NH}}_{2}C{\left({\mathrm{CH}}_{2}\mathrm{OH}\right)}_{3})$ purchased from Sigma-Aldrich (Burlington, MA, USA). All of them had a minimum purity of 99.0%. For the contact angle measurements, a set of the following liquids was applied: Milli-Q water, formamide (Acrōs Organics, 99.5%), and diiodomethane (Sigma-Aldrich, 99.0%).

### 3.2. PEEK Substrate Purification

Commercial PEEK plates were put into a 1L beaker and purified with a neutral extran (1.0 mL/100 mL of Milli-Q water), methanol, and Milli-Q water 2–3 times, until its conductivity did not exceed 5.0 μS/cm. After each liquid filling, the samples remained for 15 min in the ultrasonic bath. After this procedure, the plates were dried in the vacuum oven and then stored in desiccators.

### 3.3. Air Plasma Treatment of PEEK Surface

The air plasma (20 °C and 0.2 mbar) was applied to increase the adhesion of the PEEK surface. The optimal plasma parameters were established based on our previous research [20,30]. The plasma treatment was conducted with the aid of the microwave plasma generator (Diener Electronic, Germany). The duration of the plasma process was 60 s, the power was 460 W, and the continuous air flow was 22 sccm (standard cubic centimeters per min). The activated PEEK surfaces were further modified immediately after the plasma treatment.

### 3.4. Preparation of Chitosan (Chit), Bioglass (BG), and Bioglass-Chitosan (BG-Chit) Mixed Layers on the Plasma Activated PEEK Surface

The proper amount of chitosan was dissolved in 0.1% acetic acid (AA) to obtain the concentration of 1.0 mg/mL. The 0.2 mg/mL solution of the Chit was obtained by mixing the appropriate amount of 1.0 mg/mL Chit with 0.1% AA. The bioglass solution was prepared by mixing 0.125 g of BG with 25 mL of 0.1% AA. The mixed BG-Chit suspensions were prepared by mixing 0.125 g of BG with 25 mL of chitosan solution (1.0 mg/mL or 0.2 mg/mL). Immediately after the activation, the PEEK plates were submerged in 25 mL of the proper solution for 5 min, with continuous mixing of the solution/suspension with a magnetic stirrer. Next, the plates were dried in the vacuum oven (24 h, 20 °C) and subjected to further testing.

### 3.5. Wettability Tests and Determination of the Surface Free Energy

To study the changes of the PEEK surfaces wettability after the multi-step modification, the contact angle measurements were performed. The set of probe liquids consisted of Milli-Q water, formamide, and diiodomethane. The contact angle measuring system DGD ADR from GBX S.A.R.L was exploited. An amount of 6 μL of the test liquid was carefully placed on the PEEK surface using a microsyringe. The advancing contact angle was measured with the aid of a digital camera and the WinDrop++ software. For each sample, these measurements were conducted twice and for each tested liquid, the contact angle was examined for not less than 10 droplets on the surface. The values of the average contact angles of Milli-Q water (W), formamide (F), and diiodomethane (DM) were further used for the surface free energy calculations. 

Applying the Lifshitz-van der Waals-acid-base (LWAB) approach it was possible to estimate changes of the surface free energy of the investigated PEEK surfaces. This approach involves the values of the advancing contact angles of three liquids of different polarity and the well-established surface tensions contributions. As a result, the values of the total surface free energy ($\gamma_{s}^{tot}$) and its components, apolar Lifshitz-van der Waals ($\gamma_{s}^{LW})$, electron-donor ($\gamma_{s}^{-})$, electron-acceptor $\left(\gamma_{s}^{+}\right),$ and acid-base $\left(\gamma_{s}^{AB}\right)$, were obtained [50]. For the probe liquids, the following values of the surface tension and its components were used for calculations (Table 1):

### 3.6. Fourier Transform Infrared Spectroscopy (FTIR) Analysis

The FTIR analysis was used for the initial assessment of the surface chemistry of the modified PEEK plates. The samples were studied under the same conditions with the FTIR spectroscope equipped with the attenuated total reflectance (ATR) accessory. The deuterated triglycine sulfate (DTGS) KBr detector was used for obtaining the IR spectral data. The spectra were recorded in the range of 4000–400 cm^−1^ with 256 scans with a resolution of 4 cm^−1^ and the optimal signal-to-noise ratio. There were at least two samples of each surface and 3 measurements on each sample. The data collection and analysis were performed with Omnic 12 (Thermo Fisher Scientific, Madison, WI, USA). For the analysis, three single spectra were recorded at different points of the investigated samples, averaged into one spectrum representative for a given sample, and normalized to the band at 2918 cm^−1^ assigned to the C−H stretching. The baseline corrections were multipoint and applied at 4000, 1260, 560, and 400 cm^−1^.

### 3.7. Scanning Electron Microscopy (SEM) Analysis

Surface imaging was conducted by means of the high-resolution scanning electron-ion microscope (SEM) Quanta3D FEG (USA). The detection was made using the Everhart-Thornley detector (ETD) with a voltage of 5 kV. The pressure in the chamber was always below 7 × 10^−3^ Pa. There were at least two samples of each surface measured and the magnification varied from 100× to 200,000×. The best images with the magnifications of 500–50,000× were selected for presentation. Since the PEEK polymer is a weak conductor, the samples were sputtered with a thin layer of silver and palladium just before the measurement with the Leica EM SCD 500 (Vienna, Austria) sputtering machine.

### 3.8. Time of Flight Secondary Ion Mass Spectrometry (TOF-SIMS) Analysis

The TOF-SIMS spectra were obtained by means of the TOF-SIMS.5 instrument (ION- TOF GmbH, Münster, Germany). The primary ion source of $Bi^{+}$ was used as a primary beam for the analysis (30 kV, cyclic time 100 µs, primary beam current 1.2 pA) in the positive and negative Spectrometry mode. The analysis area was 200 µm × 200 µm, with 256 × 256 resolution and 1 shot/pixel. All the measurements were performed under static mode (dose no larger than 1 × 10^12^ ions/cm^2^). The surface potential equal to −430 V was applied to eliminate sample charging. Moreover, for depth profiles, a gun cluster ion beam (GCIB) at 7.5 keV was used for sputtering. For this purpose, the measurements were taken in the not-interlaced mode, including one analysis scan ($Bi^{+}$) and, subsequently, three sputtering scans (GCIB). The scanned area for the in-depth profile measurements was 100 µm × 100 µm, while the sputtering area was 300 µm × 300 µm. For the acquisition of chemical maps, the Fast Imaging mode was applied (cyclic time 100 µs, primary beam current 0.4 pA) that allowed the obtaining of a lateral resolution below 0.15 µm. The analysis area was 200 µm × 200 µm or 400 µm × 400 µm with a resolution 512 × 512 pixels. For the analysis, there were at least two samples of each surface and 3 measurements of each sample.

The post-processing data analysis was conducted using the SurfaceLab 6.7 software (ION-TOF) and Origin 2019 (OriginLab, Northampton, MA, USA). The negative spectra were recorded and calibrated using the positions of $CH^{-}$, $CH_{2}^{-}$, and $CH_{3}^{-}$, while the positive mode used the $CH^{+}$, $C_{2}H_{3}^{+}$, and $C_{2}H_{5}^{+}$ fragments. All intensities were normalized to the total intensity.

### 3.9. Bioactivity Tests Applying Incubation in the Simulated Body Fluid (SBF)

The simulated body fluid was prepared using a widely known procedure established by Kokubo and Takadama [43]. After preparation, the SBF was poured into the plastic bottle and stored in the fridge (8 °C). For the incubation purposes, 50 mL of SBF was poured to the plastic containers (7 cm height and 3 cm diameter) with a tight closure. Next, the dried PEEK samples were put into the containers with SBF and placed in the laboratory dryer at 37 °C for 21 days. Two samples of each surface were simultaneously incubated (in different bottles). After, the PEEK plates were gently rinsed with the Milli-Q water, dried, and then subjected for further analyses.

## 4. Conclusions

The activation of the PEEK polymer surface with the air plasma allowed the immobilization of bioglass, chitosan, and bioglass-chitosan mixed coatings on its surface, which was confirmed by the FTIR, SEM, and TOF-SIMS analyses. Chitosan was accumulated partially on both the PEEK and bioglass surfaces depending on its concentration. This affected the PEEK wetting properties, as well as changed the proportions of the surface free energy components, mainly related to the interactions through polar groups ($\gamma_{s}^{AB}$ and $\gamma_{s}^{-}$). The creation of apatite structures was observed for the PEEKp and bioglass-containing samples after immersing in SBF. The amount of apatite was also dependent on the chitosan concentration.

We believe that the discussed creation of the hybrid coatings based on chitosan and bioglass on the activated PEEK surface can contribute definitely to the development of a largely biocompatible material that combines various features, such as antibacterial or anti-inflammatory properties. For further perspectives, more specialized experiments involving the cell lines (e.g., MG63), as well as bacteria, should be conducted to confirm the predicted biocompatibility and antibacterial properties.

## Figures and Tables

**Table 1:** Values of surface tension and its components of the used testing liquids.

Probe Liquid	$\gamma^{tot}\;[\mathrm{mJ}/m^{2}]$	$\gamma^{LW}\;[\mathrm{mJ}/m^{2}]$	$\gamma^{AB}\;[\mathrm{mJ}/m^{2}]$	$\gamma^{+}\;[\mathrm{mJ}/m^{2}]$	$\gamma^{-}\;[\mathrm{mJ}/m^{2}]$
Water	72.8	21.8	51.0	25.5	25.5
Formamide	58.0	39.0	19.0	2.3	39.6
Diiodomethane	50.8	50.8	0	0	0

## Data Availability

Data available on request.
